# Design and construction of a spin-wave lens

**DOI:** 10.1038/srep33169

**Published:** 2016-09-21

**Authors:** Jan-Niklas Toedt, Mark Mundkowski, Detlef Heitmann, Stefan Mendach, Wolfgang Hansen

**Affiliations:** 1 Institute of Nanostructure and Solid State Physics, University of Hamburg, Jungiusstrasse 11, D-20355 Hamburg, Germany

## Abstract

In this work, we present the focusing of a Damon-Eshbach wave in a Ni_80_Fe_20_ film by a shaped, discrete transition of the film thickness. We devised an algorithm to determine the required shape of a spin-wave lens. Due to the anisotropy three geometries qualify as plano-convex lenses. One lens geometry has been realized experimentally and the emitted spin-wave pattern is investigated by time-resolved scanning Kerr microscopy.

Control of spin-wave propagation is crucial for applications such as spin-wave based logic gates^1^. Related investigations were mainly concentrated on guidance^2^,^3^ and manipulation^4^,^5^ using ferromagnetic micro stripes. However, there are only a few studies on controlling spin-wave propagation in unconfined ferromagnetic layers. These studies revealed promising concepts to accomplish this task: The intrinsic effects of the anisotropic spin-wave dispersion in in-plane magnetized media were utilized to control the propagation of spin waves^6^,^7^ and patterned films were used to diffract spin waves^8^,^9^. Refraction of spin waves was proposed by an altered magnetic configuration^10^ and most recently realized by an alteration (step-edge) of the thickness of a Ni_80_Fe_20_ film^11^. An apparent application of refraction is the construction of a spin-wave lens. Csaba *et al*. simulated a lens for spin waves based on the alteration of a magnetic field^12^, however the lens was designed in a regime for which spin waves exhibit almost isotropic propagation characteristics, circumventing the, in general strong, anisotropy of the spin-wave propagation. This anisotropy urges the need for an appropriate lens geometry.

Subject of this paper is the realization of a spin-wave lens optimized for a Damon-Eshbach type spin wave based on the alteration of the thickness of a Ni_80_Fe_20_ film. We use a single refractive interface to achieve focusing, analog to a plano-convex lens. Due to the anisotropy of spin waves with respect to the magnetization direction, an according lens geometry has to be found. By employing an iterative algorithm we identify three possible geometries for a focusing lens. The existence of more than one geometry is attributed to the anisotropy of the dispersion relation. We have experimentally realized one of the lens geometries using lithography and find good agreement with our model calculation used for the lens design. In particular, the resulting focal spot is found to be of similar size as the wavelength of the spin wave.

## Basics of Spin-wave Refraction

**Figure 1:**
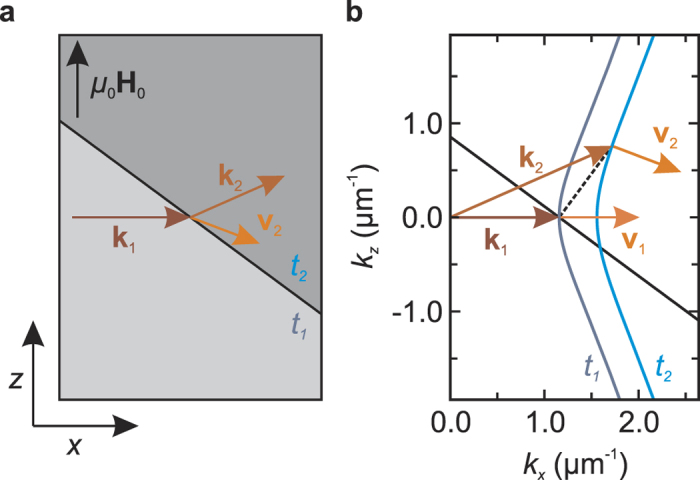
(**a**) Illustration of the refraction of a spin wave incident on a straight boundary of two Ni_80_Fe_20_ films with different thicknesses. The wave vectors of the incident wave and the refracted wave are marked as **k**_1_ and **k**_2_ respectively. (**b**) Isofrequency curves of spin waves in two differently thick (*t*_1_ > *t*_2_) Ni_80_Fe_20_ films. The transferred wave vector Δ**k** due to refraction and the orientation of the step edge are marked by the dashed and solid black line, respectively. The group velocities are marked by **v**_1_ and **v**_2_. The solid black line denotes the direction of the edge.

We will give a short introduction to the refraction of spin waves by a singular variation of the film thickness. We will first consider the example of a monochromatic plane spin wave impinging on a straight boundary of two regions with different thicknesses. Figure 1(a) depicts the considered scenario: A plane Damon-Eshbach type wave with wave vector **k**_1_ in a *t*_1_ thick film impinges on a straight boundary of a *t*_2_ < *t*_1_ thick film. The change in the film thickness alters the dispersion relation for spin waves^13^, hence causing refraction at the interface of the two regions. We point out that the refracted spin wave has now a mixed Damon-Eshbach and Backward-Volume character. It is helpful for the description of refraction to consider the isofrequency curves (i.e., the curves of all wave vectors with the same frequency) in reciprocal space, derived from the dispersion relation for spin waves in thin films^13^. In Fig. 1(b) a cutout of a isofrequency curve close to the origin is shown containing the regime of wave vectors used in our experiment. In this example the incident wave is of Damon-Eshbach type (**k**_1_ ⊥ **H**_0_). The resulting refracted wave vector is marked as **k**_2_. Here, the anisotropy renders the concept of a refractive index futile, since the refractive index would vary in dependence of the wave-propagation direction. Instead it is necessary to consider a generalized form of Snell’s law^14^: The wave number parallel to the boundary remains conserved^10^ and the difference of the wave vector Δ**k** = **k**_1_ − **k**_2_ (marked by the dashed line) is therefore perpendicular to the boundary. For curved structures this description decreases in accuracy with increasing curvature (smaller structures). In the considered regime of spin-waves the dispersion relation is strongly anisotropic, i.e. the wavelength of a Damon-Eshbach type wave is about 30 times longer than the wavelength of a Backward-Volume mode. Hence, the conservation of the tangential wave component remains a good description for refraction at structures much smaller than in the isotropic case.

## Lens Design

**Figure 2:**
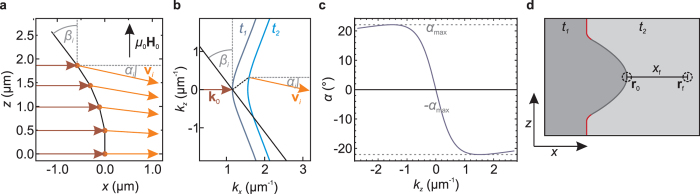
(**a,b**) Illustration of the algorithm used to calculate the lens shape. A planar spin wave with wave vector **k**_0_ in the layer with thickness *t*_1_ is assumed to impinge from the left. The orange dots mark the iteration points at which the needed angle *α*_*i*_ of the group velocity **v**_*i*_ is calculated. (**c**) Angle of the group velocity on the isofrequency curve valid for *t*_2_ as a function of *k*_*z*_. (**d**) Final shape of the designed lens marked in red are parts of the structure which do not act as lens.

We now consider a lens with a curved boundary line. The shape of the lens is calculated in an iterative fashion, assuming a monochromatic, planar Damon-Eshbach type spin wave as incident wave. Figure 2 illustrates the algorithm used to find the lens shape, which will be explained in the following. As in the previous example we consider an abrupt interface of two films with different thicknesses; we consider the incident wave to impinge from the thicker layer (*t*_1_ > *t*_2_). As axis of incidence we chose the *x* axis. The focal spot is at **r**_*f*_ = (*x*_*f*_, 0). To achieve focusing the spin wave has to be refracted in such a way that the resulting group velocity points in the direction towards the desired focal spot. This angle is determined by


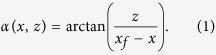


The contour of the lens will be approximated by linear parts of equal length *l*. The determination of the lens shape is started at **r**_0_ = (0, 0). Due to symmetry it is sufficient to calculate the lens shape for *z* ≥ 0. At the starting point of the iteration, no change of the direction of the group velocity is required, which is satisfied by a vertical segment of the boundary. The first segment of the lens hence stretches from **r**_0_ = (0, 0) to **r**_1_ = (0, *l*). At **r**_1_ the required angle *α*_1_ of the refracted wave’s group velocity determined by Eq. (1) is *α*_1_ = arctan(*l*/*x*_*f*_). The isofrequency curve belonging to the region with thickness *t*_2_ is scanned numerically for this value and the according wave vector **k** is returned. As the incident wave is a Damon-Eshbach spin wave with wave vector **k**_0_ = (*k*_0_, 0), the required change of the wave vector by refraction is given by


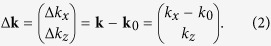


The aforementioned condition of the boundary being perpendicular to Δ**k** can be used to determine the required tangent of the lens, yielding the relation


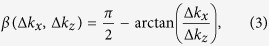


where *β* is the angle between the tangent of the boundary and the *z* axis. The next point of evaluation is at a distance of *l* following the calculated tangent. The procedure is iterated in this fashion.

Figure 2(c) depicts the angle of the group velocity *α* on the isofrequency curve at the thickness *t*_2_ as a function of *k*_*z*_ for the parameters in the experiment, as described later. In the considered regime of Damon-Eshbach like spin waves *α* is constrained between *α*_max_ and −*α*_max_. This defines the terminus of the iteration; the iteration is aborted, when the required absolute value of *α* exceeds *α*_max_. The lens contour merges into a line aligned in *z* direction with an arbitrarily smoothed transition. The resulting shape is depicted in Fig. 2(d). The designed lens consists of only a single transition between different media in contrast to classical convex lenses. A straight transition from *t*_2_ to *t*_1_ in front of the lens would complete the lens as analog to a plano-convex lens. Here we omitted this first transition, as it does not alter the wave incident onto the curved boundary.

**Figure 3:**
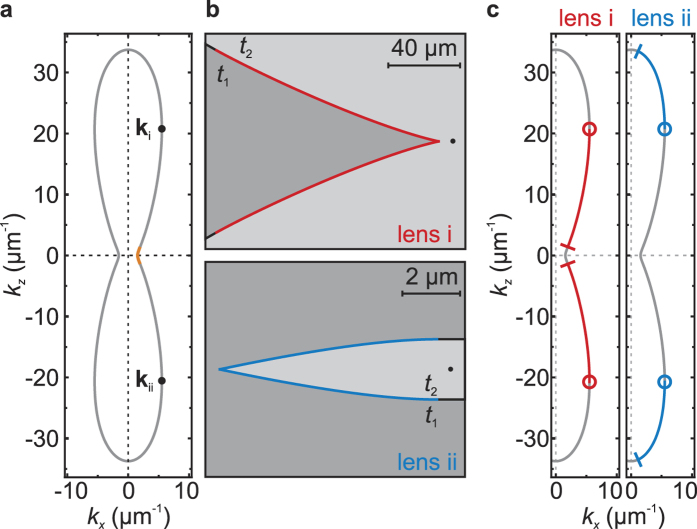
(**a**) Isofrequency curve for *t*_2_ at *μ*_0_*H*_0_ = 10 mT and *f* = 5 GHz. The orange section repesents the wave vectors emitted be the derived lens. Wave vectors with group velocities parallel to that of the Damon-Eshbach direction are indicated as *k*_a_ and *k*_b_. (**b**) Lens designs resulting in the choice of *β*_a_ (top) and *β*_b_ (bottom) as starting angles. The focal spot is marked by a black dot. The black lines mark sections of the lenses which do not contribute to focusing. (**c**) Wave vector intervals emitted by the kinked lenses. The wave vectors emitted at the lens center and termini are marked by circles and dashes, respectively.

For the previously described lens design an edge along the *z* direction was chosen for *z* = 0. However, the isofrequency curve exhibits not only one wave vector with group velocity parallel to the Damon-Eshbach (*x*) direction but two additional ones. Those are highlighted in Fig. 3(a) as **k**_a_ and **k**_b_. The tilt angles *β*_a_ and *β*_b_ to acquire these wave vectors are thus also valid edge orientations for *z* = 0 and result in two additional possible lens geometries. Due to the axis symmetry of the lens a starting angle *β*_0_ ≠ 0 results in a kink at the lens center, hence we will refer to these designs as kinked lenses and to the previously derived design as smooth lens. Figure 3(b) shows the two alternative lens designs obtained with the parameter set used for the previously described lens design. The top panel depicts the lens with *β*_a_ as starting edge angle for the upper half (hereafter lens i) and the bottom panel depicts the lens with *β*_b_ as starting edge angle (hereafter lens ii). Please note the different scale of the lenses. Again, the spin wave is incident from the left, the focal spot is marked by a black dot. Marked in black is an arbitrary continuation of the thickness step after the lens termini is reached, similar to Fig. 2(c). Figure 3(c) depicts the sections of the isofrequency curve accessed by the different lenses. The wave vectors generated at the termini and at the lens center are respectively indicated by dashes and circles. While the smooth lens emits a single interval/section of wave vectors, the emitted *k* spectra of the kinked lenses miss a section of wave vectors around the axis of incidence, not unlike to the technique of dark-field microscopy.

The wave vectors contributing to the focal spot of the kinked lenses are much larger than those of the smooth lens and thus the achievable focal spot size should be in principle much narrower. However, there are a series of deficiencies inherent to the kinked lens which will be discussed in the following. Foremost, the large wave vector difference upon refraction would lead to a considerable reflection at the lens surface. This could be circumvented by choosing a film thickness gradient rather than a step in thickness. The very large cross section of lens i would gather a considerably larger incident spin-wave power, somewhat alleviating the issue. Secondly the near right angles between the external field and the lens boundary are would induce a pronounced heterogeneity of the internal static magnetization, especially for lens ii. As the emitted spin-wave of lens i travels relatively close to the edge a large phase mismatch could be accumulated. This magnetic inhomogeneity could be averted by either choosing a small size of the thickness step or again using a continuous thickness transition. However, the comparably small group velocities associated with spin waves near the Backward-Volume regime results in shorter decay lengths for those spin waves, which would be especially detrimental for lens i due to the long distances between lens boundary and focal spot. Despite these problems, most of which are unique to spin waves, it is nonetheless remarkable, that the anisotropy of the dispersion enables the appearance of additional geometric solutions which satisfy the condition for a plano-convex focusing lens. The smooth lens was selected to be experimentally realized, since it promises comparably small reflection at the thickness transition, the lowest magnetic inhomogeneities, and emitted wave lengths in the resolvable range of the employed scanning Kerr microscope.

## Sample Design and Measurement Setup

**Figure 4:**
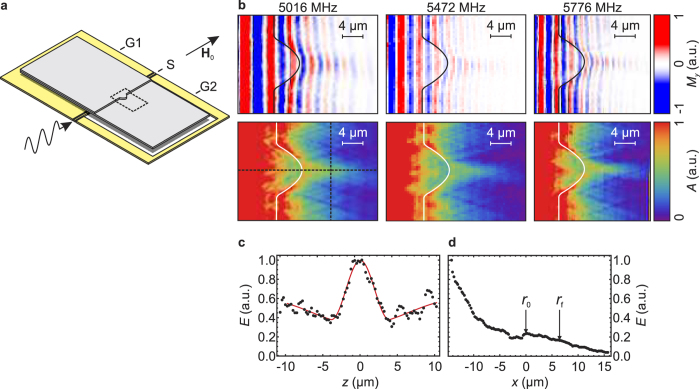
(**a**) Illustration of the experimental arrangement. G1 and G2 mark the ground lines of the wave guide. S marks the signal line of the wave guide. (**b**) Top panel: Measurements of the out-of-plane component (*M*_*y*_) of the spin-wave pattern at the lens, taken at an external magnetic field of *μ*_0_*H*_0_ = 10 mT. Bottom panel: Amplitude of the spin-wave pattern corresponding to the measurements on the left-hand side. The three patterns are recorded at different mw frequencies marked on the right hand side. (**c,d**) Scans of the amplitude at *f*_1_ = 5016 MHz along the *z* and *x* axes, as indicated by the dashed lines in (**b**).

For the experiments we prepared on a GaAs(001) substrate a coplanar wave guide consisting of a 5 nm thick layer of Chromium and a 150 nm thick layer of gold. The central, 3 μm wide signal line (S) is separated from the surrounding ground lines (G1 and G2) by a 2 μm wide gap. The wave guide is covered by a 220 nm thick insulating layer of hydrogen silsesquioxane (HSQ). On top of the HSQ layer a rectangular, 30 nm thick permalloy film was deposited. At the position of the permalloy film G2 exhibits a gap to ensure the excitation of well defined planar spin waves by the signal line (S) (cf. Fig. 4(a))^8^. A second 15 nm layer of permalloy layer was deposited onto the first permalloy film. This film was patterned by means of electron-beam lithography and lift-off method into a stripe from which the lens pokes out like shown in Fig 2(d). The film-thickness transition was measured by means of atomic force microscopy to be less than 30. The right edge of the stripe and the tip of the lens are positioned at distances from the signal of 10 μm and 13.9 μm, respectively. The thickness of the combined and the single layer are *t*_1_ = 45 nm and *t*_2_ = 30 nm, respectively.

The spin-wave pattern of the lens was investigated by means of time-resolved scanning Kerr microscopy^15^. The spin waves in the sample are excited by the alternating magnetic field of a micro-wave current fed into the signal line of the coplanar wave guide. A train of linear polarized laser pulses, emitted by a titanium sapphire laser with a repetition frequency of *f*_rep_ = 76 MHz and the central wavelength of *λ* = 800 nm is focused on the sample. The incidence is perpendicular to the sample surface. In this configuration the polarization of the reflected beam is affected by the polar Kerr effect, hence we detect the out-of-plane component of the magnetization *M*_*y*_. The reflected beam is separated into two perpendicularly polarized beams by a Wollaston prism. The separated beams are guided onto two photo diodes and the difference of the signal is measured by a Lock-In amplifier triggered onto a modulation applied to the micro wave current. The micro-wave current in the wave guide is phase locked to the laser pulses and time resolution is achieved by altering the phase *ϕ* of the exciting radio-frequency current with respect to the laser pulses. A 3-axis piezo actuator moves the sample under the focused laser beam, enabling spatial scanning of the sample. A static magnetic field is applied parallel to the signal line (S).

## Experimental Results and Discussion

We mapped the full period of the spin wave by scanning the *M*_*y*_ pattern of the spin wave at phase steps of Δ*ϕ* ≤ 18.9° governed by the resolution of the electrical delay. From this we determined the amplitude *A* (i.e. the envelope) of the spin-wave pattern at the lens. Figure 4(b) top panel shows spin-wave patterns of the lens. As an example we show measurements of *M*_*y*_ at frequencies *f*_1_ = 5016 MHz, *f*_2_ = 5472 MHz and *f*_3_ = 5776 MHz at a static field of *μ*_0_*H*_0_ = 10 mT. The bottom panel of Fig. 4(b) depicts the amplitudes of the spin-wave patterns corresponding to the measurements of *M*_*y*_ in the top panel. Both the map of *M*_*y*_ and the spin-wave amplitude exhibit a focusing behavior of the lens in a broad frequency regime. For higher frequencies the size of the focal spot decreases and it moves further to the lens. The wave length of the incident spin-wave decreases from *λ*_1_ = 5.2 μm at *f*_1_ to *λ*_2_ = 3.9 μm and to *λ*_3_ = 3.2 μm at *f*_2_ and *f*_3_, respectively. Likewise, the wave length of the spin wave refracted at the straight section of the thickness step decreases from 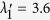 μm to 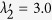 μm and to 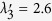 μm.

Figure 4(c) shows a scan of the envelope for the frequency *f*_1_ along the *z* direction at the focal spot as indicated by the dashed vertical line in Fig. 4(b). A clear focal peak is evident at *z* = 0 inside a much broader dip of the envelope. Both the central peak, as well as the surrounding dip can be well described as Gaussian peaks, as indicated by the red line, which is a fit based on the superposition of a narrow positive Gaussian peak and a broader Gaussian peak of negative sign. The decrease of intensity is caused by the redirection of the power of the incident spin wave towards the focal position, effectively being a shadow of the lens in the illuminating plane wave. In *x* direction, as shown in Fig. 4(d), the damping of the spin waves dominates the course of the envelope, and a focus spot is not clearly distinguishable. Only at the tip of the lens, as indicated by *r*_0_, an increase of the envelope is evident due to the reduction of the film thickness.

**Figure 5:**
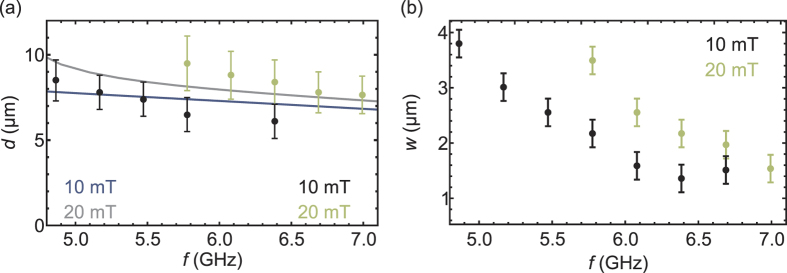
(**a**) Measured position *x*_*f*_ of the focal spot as a function of the frequency at *μ*_0_*H*_0_ = 10 mT (black dots) and *μ*_0_*H*_0_ = 20 mT (blue dots). The gray and green lines mark the calculated focal distances at 10 mT and 10 mT, respectively. (**b**) Measured width *w* of the focal spot as a function of the frequency at *μ*_0_*H*_0_ = 10 mT (black dots) and *μ*_0_*H*_0_ = 20 mT (blue dots).

To characterize the lens we investigated the position and width of the focal spot as a function of the frequency at the external fields of *μ*_0_*H*_0_ = 10 mT and *μ*_0_*H*_0_ = 20 mT. For this purpose we determined the FWHM of the focal peak in *z* direction (cf. Fig.4(c)) at all mapped *x* positions. Figure 5 shows the distance of the focal spot to the lens tip (a) and the width of the focal spot (b) as a function of the frequency; the solid line depicts the calculated focal position. Both the width of the focal spot and the distance to the lens decrease at increasing spin-wave frequencies. The frequency dependence of the focal spot has been calculated in a ray-optics approach similar to the above one used for lens design. This attempt, while obviously inappropriate for determining the shape of the focal spot due to neglection of diffraction, yet enables the determination of the focal-spot position by determining the position of minimal spread of the rays. The experimental data fit the calculation in the range of the standard deviation. The width of the focal spot is in the range of the wave length and decreases with increasing frequency, which is expected since the terminal wave vector grows with increasing frequency.

Interesting to note is the different cause of the limit of the achievable resolution compared to optical lenses. While in our case, wave vectors much larger than the wave vector of the incident spin wave are accessible, i.e. the leaving waves are much shorter, the angle of incidence at the focal spot (angular aperture) is limited due to the change in curvature of the isofrequency curve. Specifically this means, that at a fixed focal distance the lateral extent of the lens cannot be further increased, setting a limit to resolution. In contrast optical lenses can achieve much larger angular apertures but are limited in resolution as the wave length of the light remains fixed.

## Conclusion

In conclusion, we constructed an appropriately curved spin-wave lens based on refraction of a spin wave by a step edge in a permalloy. We presented an algorithm to determine the shape of a lens for a specific set of film thicknesses, external field and spin-wave frequency. This algorithm can be employed with any type of wave in media with anisotropic dispersion. Three lens geometries were identified, the appearance of the additional geometries is enabled by the anisotropic spin-wave dispersion. One of the geometries was constructed. By means of TR-SKM microscopy we experimentally demonstrated the viability of the designed spin-wave lens and determined the frequency dependency of both the focal distance and the focus width. The experimental results are in good agreement with the ray optics approach used for the design of the lens.

## Additional Information

**How to cite this article**: Toedt, J.-N. *et al*. Design and construction of a spin-wave lens. *Sci. Rep.* **6**, 33169; doi: 10.1038/srep33169 (2016).

## References

[b1] Chumak A. V., Vasyuchka V. I., Serga A. A. & Hillebrands B. Magnon spintronics. Nat Phys 11, 453–461, 10.1038/nphys3347 (2015).

[b2] Demidov V. E., Demokritov S. O., Rott K., Krzysteczko P. & Reiss G. Nano-optics with spin waves at microwave frequencies. Applied Physics Letters 92, 232503, 10.1063/1.2945000 (2008).

[b3] Vogt K. . Spin waves turning a corner. Appl. Phys. Lett. 101, 042410 (2012).

[b4] Demidov V. . Transformation of propagating spin-wave modes in microscopic waveguides with variable width. Phys. Rev. B 79, 212501, 10.1103/physrevb.79.054417 (2009).

[b5] Demidov V. E. . Excitation of short-wavelength spin waves in magnonic waveguides. Appl. Phys. Lett. 99, 082507, 10.1063/1.3631756 (2011).

[b6] Demidov V. E. . Radiation of spin waves from the open end of a microscopic magnetic-film waveguide. Phys. Rev. B 80, 014429, 10.1103/PhysRevB.80.014429 (2009).

[b7] Schneider T. . Nondiffractive subwavelength wave beams in a medium with externally controlled anisotropy. Phys. Rev. Lett. 104, 197203, 10.1103/PhysRevLett.104.197203 (2010). 20866995

[b8] Mansfeld S. . Spin wave diffraction and perfect imaging of a grating. Physical Review Letters 108, 047204, 10.1103/physrevlett.108.047204 (2012). 22400886

[b9] Perzlmaier K., Woltersdorf G. & Back C. Observation of the propagation and interference of spin waves in ferromagnetic thin films. Phys. Rev. B 77, 054425, 10.1103/physrevb.77.054425 (2008).

[b10] Kim S.-K. . Negative refraction of dipole-exchange spin waves through a magnetic twin interface in restricted geometry. Appl. Phys. Lett. 92, 212501, 10.1063/1.2936294 (2008).

[b11] Tanabe K. . Real-time observation of snell’s law for spin waves in thin ferromagnetic films. Applied Physics Express 7, 053001, URL http://stacks.iop.org/1882-0786/7/i=5/a=053001 (2014).

[b12] Csaba G., Papp A. & Porod W. Spin-wave based realization of optical computing primitives. Journal of Applied Physics 115, 17C741, 10.1063/1.4868921 (2014).

[b13] Kalinikos B. A. & Slavin A. N. Theory of dipole-exchange spin wave spectrum for ferromagnetic films with mixed exchange boundary conditions. Journal of Physics C: Solid State Physics 19, 7013, URL http://stacks.iop.org/0022-3719/19/i=35/a=014 (1986).

[b14] Slawinski M. & Slawinski R. Energy partition at the boundary between anisotropic media; part one: Generalized snell’s law. CREWES Research Report 6, 9 (1994).

[b15] Freeman M. R. & Smyth J. F. Picosecond time-resolved magnetization dynamics of thin-film heads. J. Appl. Phys. 79, 5898–5900, 10.1063/1.361896 (1996).

